# Performance analysis of improved path loss models for millimeter-wave wireless network channels at 28 GHz and 38 GHz

**DOI:** 10.1371/journal.pone.0283005

**Published:** 2023-03-21

**Authors:** Tolulope T. Oladimeji, Pradeep Kumar, Mohamed K. Elmezughi

**Affiliations:** Discipline of Electrical, Electronic and Computer Engineering, University of KwaZulu-Natal, Durban, South Africa; Vinnytsia National Technical University, UKRAINE

## Abstract

The importance of the path loss in millimeter wave channel propagation cannot be taken for granted in terms of deployment, design, performance assessment, and planning. The path loss helps to determine the network’s geographic coverage. Although many path loss models, including statistical and empirical models based on measurement and linear regression, have been proposed by various researchers, high fidelity is required to determine the performance of the wireless network’s channel. This research validates the improved version of the well-known close in (CI) and floating intercept (FI) path loss models at frequency bands of 28 and 38 GHz. The measurement surroundings comprised of an enclosed passageway with vertical-horizontal (V-H) and vertical-vertical (V-V) antenna polarizations. One of the key findings of this study is that the enhanced versions of these models typically perform better in terms of consistency than the standard models thereby justifying their high accuracy level. The improved versions of the CI and the FI models demonstrate a significant improvement for various antenna polarizations. The mean prediction error (MPE) and standard deviation error (SDE) also show how precisely and accurately the improved models predict the path loss. Additionally, the improved models provide the reasonable responsiveness and uniformity of the parameters with the change in the antenna polarization and lower the shadow fading’s standard deviation in LOS as well as NLOS situations. The results confirm that the modified versions of CI and FI models predict path loss better in an enclosed environment for 5G networks.

## 1. Introduction

In the last few years, there has been a tremendous amount of change in the field of telecommunications. There is a need for general improvement of today’s mobile communication system in order to meet future expectations and challenges [1, 2]. 5G mobile networks are widely expected to address some important issues, including higher data rates, widespread device connectivity, larger capacity, low cost and dependability [2–8]. However, the anticipated increase in traffic may be accommodated by increasing link capacity, opening up more spectrums, and massively increasing the density of tiny cells [9].

**Fig 1:**
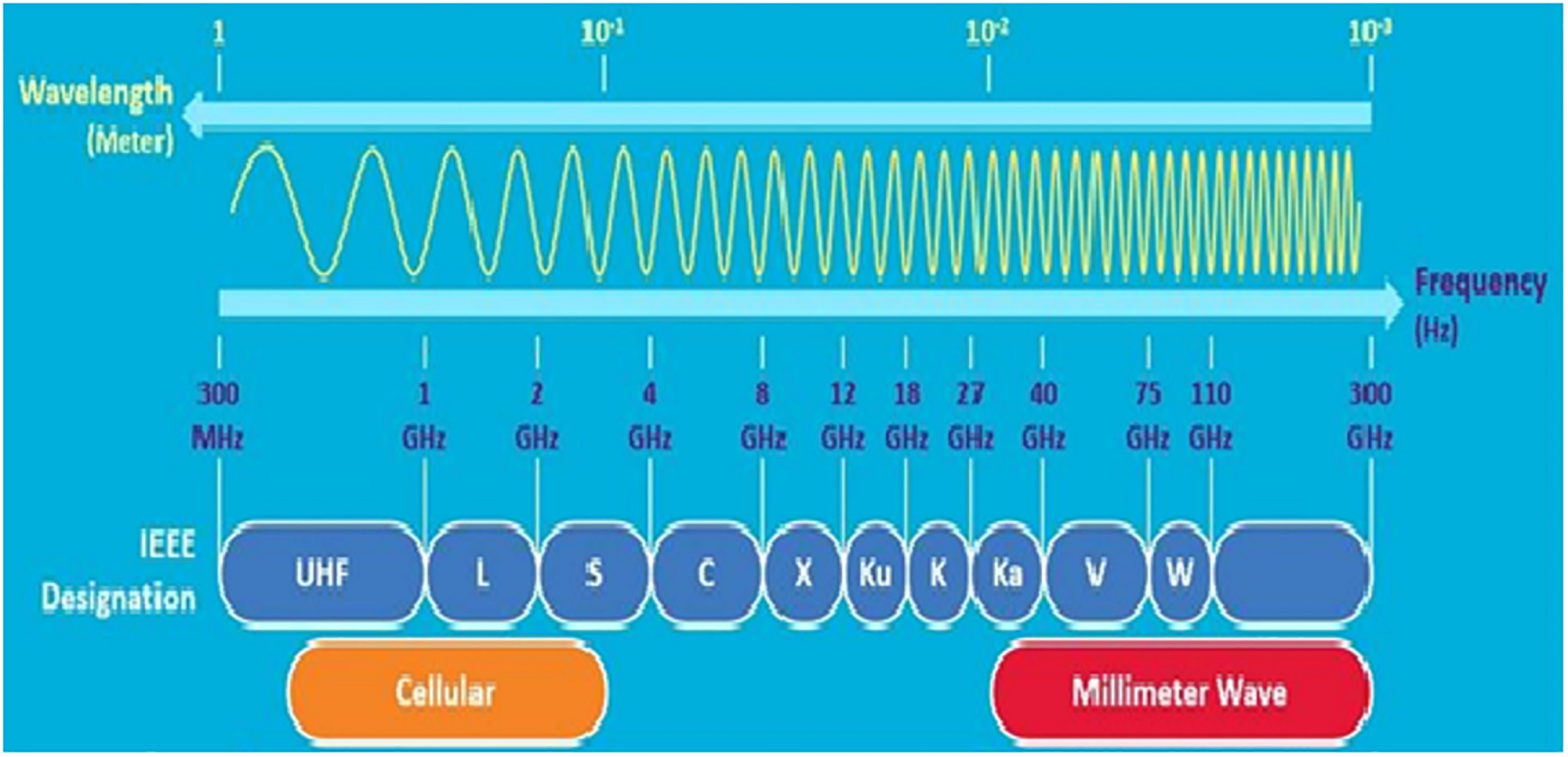
MmWave frequency spectrum band [12].

In order for the wireless industry to meet the demands associated with 5G networks, there is a need for the millimeter wave (mmWave) frequency band (FB). This will help in developing previously untapped FB as well as multi- Giga bit per seconds (Gbps) data rates for mobile devices. MmWave technology is required because FB ranges below 6 GHz lack the bandwidth capacity required for 5G wireless systems to achieve maximum data transmission rates of multiple Gbps [10, 11]. The majority of the mmWave FB bands are located between microwave and infrared waves, as shown in Fig 1 [12].

Furthermore, the business world has recognized the value of wireless communication, particularly for gigabit speeds, longer range connectivity, and high-quality multi-media, voice, as well as data services [1, 10, 13–16]. Because these bands have the potential for the implementation of 5G wireless networks and meeting future demands, there has been an increase in recent years in research into the mmWave FB (30–300 GHz) [7]. In the mmWave FB, massive contiguous blocks of raw bandwidth are available, allowing for increased data flow for various multimedia services. The upper centimeter wave (cm wave) bands of the licensed spectrum will remain accessible to 5G technology [1, 10, 17–22].

The abundance of untapped spectrum benefits mmWave bands. Along with the wider bandwidth, running at a higher FB, comes the following benefits: intrinsic beam forming improves wireless signal (WS) strength and spectral efficiency because (i) antennas can achieve stronger directivity at higher FBs, and (ii) smaller beams allow for greater node mobility. Despite these advantages, the applicability of these bands must be determined by classification of WS transmission behavior. MmWave bands exhibit distinct electromagnetic propagation with varying reflection and diffraction characteristics. They also have higher absorption losses [23–25]. The cell radius for mmWave bands, on the other hand, is significantly smaller (a few hundred meters), because the environment in which the WS travels has a significant effect on its ability to spread. Furthermore, the penetration loss in solid materials has a significant impact on the mmWave frequency spectrum. It is critical to accurately understand and characterize the behaviors of the mmWave FBs in a variety of indoor and outdoor locations and scenarios in order to accurately analyze performance and ensure the safe deployment of 5G cellular systems [10, 26–31].

Numerous studies have been conducted on this topic in order to make predictions about the behavior of a wireless channel using models based on theories. When a communication system transmits and receives WSs, the signal strength decreases as it travels over the wireless communication channel. Path loss (PL) is a measurement of the degradation of WSs sent over a long distance in LOS as well as NLOS scenarios. PL is an important factor that must be predicted accurately for proper system design and link budget evaluation. Furthermore, it generates radio channel parameters that have been empirically averaged across both space and time. To accurately explain the attenuations in WS levels, researchers must develop more precise PL simulation models that can successfully fit real quantitative measurements obtained under various interior and outdoor conditions over a wide FB. Because of the high responsiveness of the WSs at these radio frequencies to the transmission procedures in the signal propagation process, conventional models are unable to serve as precise models for the SHF, mmWave, and far above FBs [32–35].

The measurement of this study was done in LOS as well as NLOS scenarios at 28 and 38 GHz FBs in order to evaluate CI and FI PLMs in an interior passage. The measurement was done on the fifth (5^th^) floor of the Electrical, Electronic and Computer Engineering (EECE) Department on the Howard Campus of University of KwaZulu-Natal (UKZN) in Durban, South Africa. Despite the fact that Elmeguzi and Afullo [32] proposed this improved version of CI and FI PLMs. The proposed models perform well in terms of precision of matching measured data obtained during measurement settings at FBs of 14, 18, and 22 GHz for only V-V antenna polarization. However, this model has not been validated in higher FBs or with different antenna polarizations. To the best of our knowledge, there is a gap in the literature that justifies the accuracy and stability of these proposed models by [32] in the modeling and characterization of single frequency path loss models (PLM) that consider two antenna polarizations. The work aims to test the accuracy, stability, and sensitivity of these models by using measured parameters collected in an enclosed corridor. The shadow fading standard deviation (SFSD), which is the foundation for high precision PL prediction in both LOS and NLOS scenarios, is also examined. Despite this extensive study by [32], it was discovered that at the same antenna height (for 14, 18 and 22 GHz) for both the transmitter and the receiver, there was a noticeable decrease in path loss exponent and a slight increase in shadow fading standard deviation for the NLOS scenario, but when the antenna heights are not of the same height, the two major metrics (path loss exponent and shadow fading standard deviation) increase. Nevertheless, as the frequency increases, the effect of the antenna height difference has little effect on the value of the path loss exponent and the standard deviation of shadow fading. When using two different antenna heights, several authors in [8, 10, 11, 13, 17, 19, 21, 23, 28, 30, 31, 33, 36–41] have adopted the use of higher values of antenna heights at the transmitting end of the measurement set up. However, in this study the analysis of the path loss prediction models for the higher height of the antenna at the receiving end is presented. The analysis of V-V and V-H antenna polarizations is also presented in this study. The following are the remaining sections of the study: The related works are presented in Section 2, while the measurement campaign details and large-scale PLMs are presented in Section 3. Section 4 contains the findings and discussions, while Section 5 presents the conclusion of the work.

## 2. Related works

The mmWave spectrum’s ability to produce high throughput in the Gbps spectrum, which is required for 5G systems, has been demonstrated. However, the environment in which the WS is transmitted has a significant effect on mmWave propagation. Furthermore, penetration loss through solid objects has a significant impact on WSs in the mmWave frequency range [26, 29]. The propagation scenarios as well as the FB considered have a significant impact on the accuracy of these models. Many models proposed by researchers to improve delay time, output, and path loss exponent (PLE) performance have a distinct application that still has a long way to go in terms of distance and frequency of application [36, 37]. An experiment on ultra-wideband propagation was carried out at New York University in an enclosed office setting. The statistics of large-scale PL were calculated using the experiment’s findings in both existing and upcoming applications. The tests were carried out in an enclosed structure under LOS and NLOS conditions using directional antennas at FBs of 28 and 73 GHz. The experiment revealed that simple CI and FI models can accurately predict PL in mmWave indoor wireless channels on a large scale (with distance and frequency) using only one or two functions related to transmitted power [38]. Maccarthney et al. presented some omnidirectional propagation data captured in New York Downtown metropolis [38] to ensure the accuracy and effectiveness of the CI PLM.

Sun et al. investigated propagation PLMs for 5G urban micro and macro-cellular situations using the CI and ABG as large scale PLMs. Data were collected over the course of about 20 measurement procedures that ranged in distance from 5 to 1429 m and used FBs ranging from 2 to 73.5 GHz. An examination of the research findings reveals that the CI model’s simulation accuracy performs better than the alpha-beta-gamma (ABG) model. In comparison to the latter, the former provides more consistent and tolerable performance across all FBs and distance ranges investigated during the study. To improve accuracy, the CI model, which is simple to use over a wide FB, must simply be modified [39]. The close in model with frequency-weighted PLE (CIF), the close in model with free reference distance, and the ABG were compared in a variety of data sets with distances ranging from 4 to 1238 m and FBs ranging from 2 to 73 GHz. Urban microcells, malls, and an indoor work environment were among the scenarios. While the ABG model (four parameters considered) under-predicts path losses near the Tx and over-predicts PLs far from the Tx, the CI (2 variables considered) and CIF (3 variables considered) models have better goodness of fit and more stable component behavior. This discovery holds true across all distances and FBs investigated [40].

In their study on indoor 5G 3rd Generation Partnership Project (3GPP) like channel models for workplace and commercial settings, Haneda et al. used both current and historical measurements of channel propagation for FBs up to 100 GHz. It was discovered that as frequency increased, so did penetration loss. According to the UMi and Uma models [41], the indoor channels are more frequency dependent than the outside channels.

At 28 and 38 GHz, measurements were taken in New York, Austin, and Texas as part of an empirically-based large scale propagation PL modeling for 5G mobile systems scheduling in the mmWave band. The portion of coverage increased significantly when the antenna was aimed in the optimal direction towards both the mobile and the base station. As a result, there are fewer 5G base stations, and interference is reduced [42]. The Rural Macrocell PLMs for mmWave wireless network analysis, which is part of a larger research project, provides a thorough understanding of the existing 3GPP, RMa LOS, and NLOS PLMs in the FBs of 0.5 to 100 GHz. In a rural location with good weather, directional horn antennas (HA) were used for a real-time measurement campaign using the CI and CIH model components. Even beyond the first meter of propagation distance, the observed data confirms the CIH model’s correctness, dependability, and frequency dependency [43].

The characteristics of propagation channels in the FBs of 6.5, 10.5, 15, 19, 28, and 38 GHz were further investigated in an indoor setting using a measurement campaign spanning 4,000 power delay profiles and employing a directional HA as a Rx and an omnidirectional antenna as a Tx section. The frequency attenuation model is a novel PLM that takes both distance and FB into account. RMS delay spread and dispersion factor values for this model demonstrate its simplicity, lower PLEs, and good RMS delay spread and dispersion factor values [44].

The majority of propagation models currently in use for any FB below 6 GHz are not suitable for mmWave PL modeling, nor are they suitable for any FB above 6 GHz. This is due to the obvious disparity in WS propagation. A measurement campaign was conducted indoors at Malaysia’s University of Technology (UTM Malaysia). PL analysis of single and multi-frequency signals was performed in applications utilizing omnidirectional and directional antennas. It was discovered that using a less complex model approach with only one PLE parameter (n) that is dependent on transmitted power makes modeling PL on a large scale with respect to distance easier [45]. In contrast, using a model that is not dependent on transmitted power may necessitate more variables, making the modeling complex. The examination of these PLMs demonstrates the need to develop a model that is more effective than the fundamental models, such as the CI, FI, and ABG, at reducing PL of the propagated SIG from the Tx to the Rx [45].

In [32], Elmezughi and Afullo proposed an improved model for improving the accuracy of CI and FI model standards. This enhanced model was used for FBs 14, 18, and 22 GHz taking into account wave-guiding effects, which are most common in enclosed indoor spaces like hallways, as well as propagation processes like reflections and diffractions. Because they are appropriate, specific PLMs, such as the ABG, CI, and FI models, have recently received the most attention in research aimed at describing and modeling the wireless channel [32]. In the research studies [33, 39, 41–43, 46–51], more sophisticated models based on a wide range of other precepts can be discovered.

In addition, tests for 28 GHz reflection and penetration loss were performed in buildings in various areas of New York City [49]. The results show that a three-wall office complex has a significant absorption loss of 45.1 dB. Furthermore, whereas exterior tinted glass had absorption loss of 40.1 dB, interior non-tinted glass had an absorption loss of 3.9 dB. The authors of [52] performed a measurement in a lab using 28 and 82 GHz FB, as well as an anechoic chamber, to determine the qualities of the link in the mmWave bands. Hindia et al. measured an established channel model inside an outdoor area for single and multi-frequency models, as well as proposing a novel model [53]. The authors of [46, 49, 54] conducted measurements at 28 and 73 GHz in an interior office using omnidirectional and directional HAs. Measurements and simulations were carried out at FBs of 28, 38, and 73 GHz in order to assess and estimate a number of parameters as well as classify the transmission channel in CI and ABG PLMs [55, 56]. Zwick et al. measured several wideband link statistics at 60 GHz using heterodyne and channel bandwidth of 500 MHz. They used omnidirectional antennas for close-quarters distances in a number of apartments and combined the measured data (for an overall bandwidth of 5 GHz) [57].

## 3. Measurement campaign and environment

**Fig 2:**
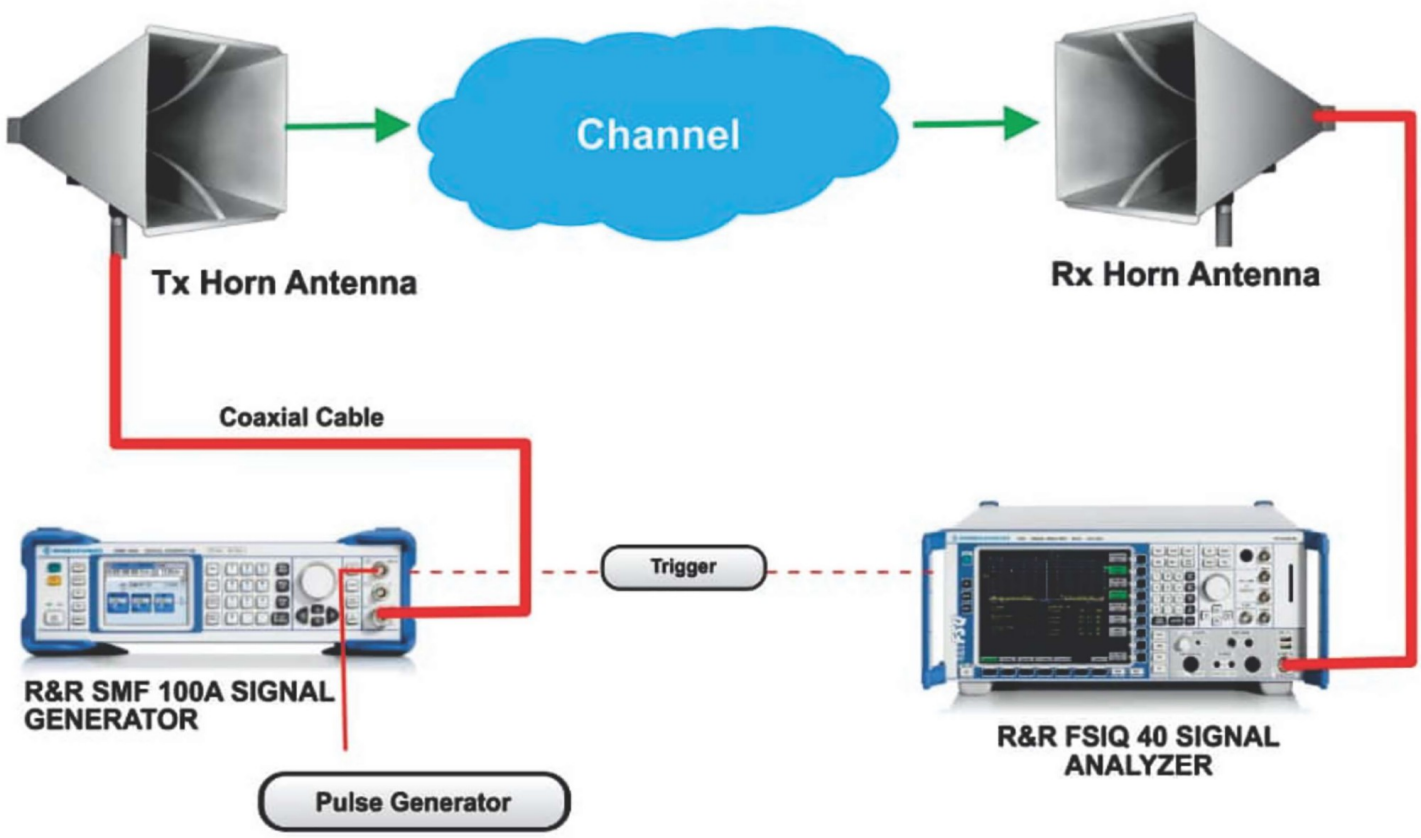
The channel sounder’s architecture.

**Fig 3:**
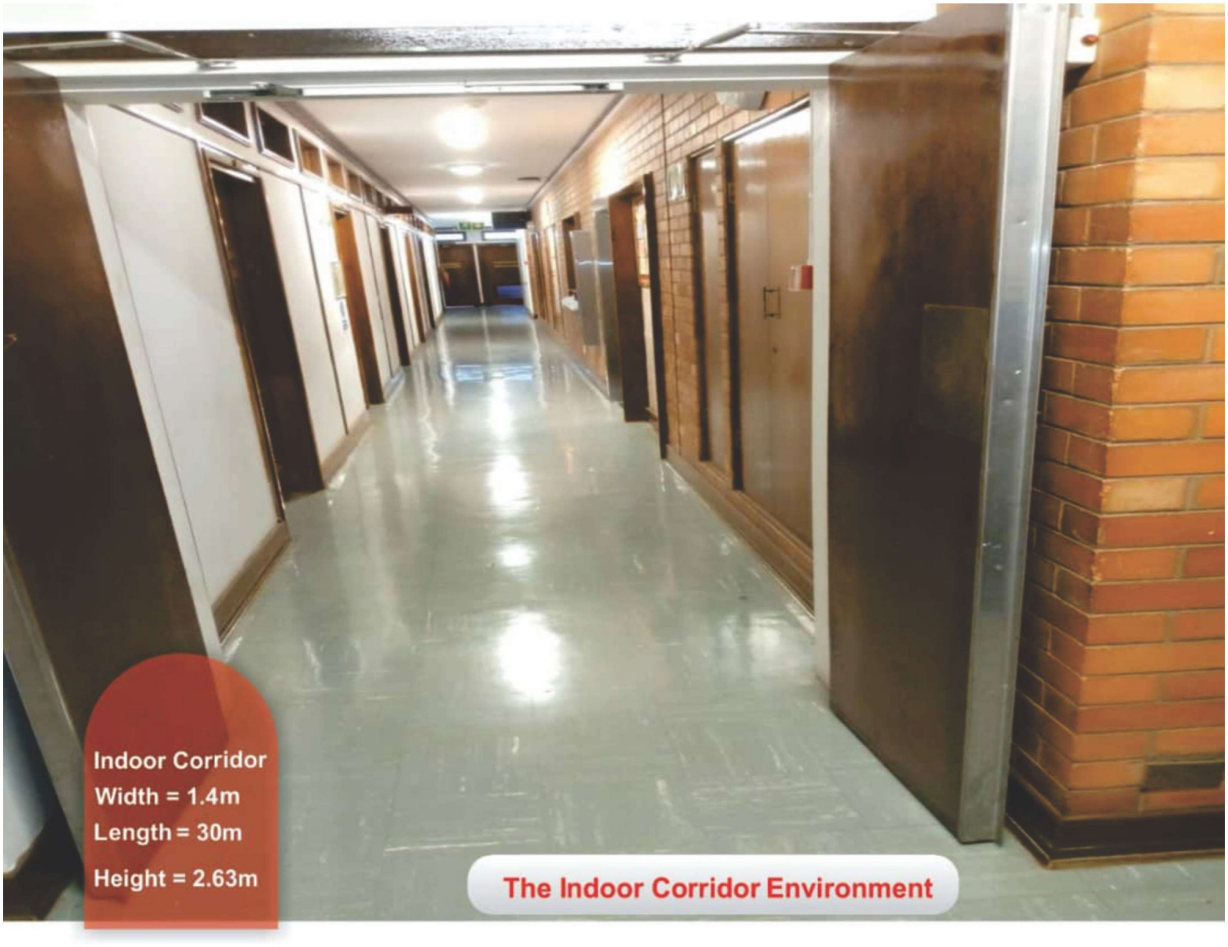
The indoor corridor environment.

**Fig 4:**
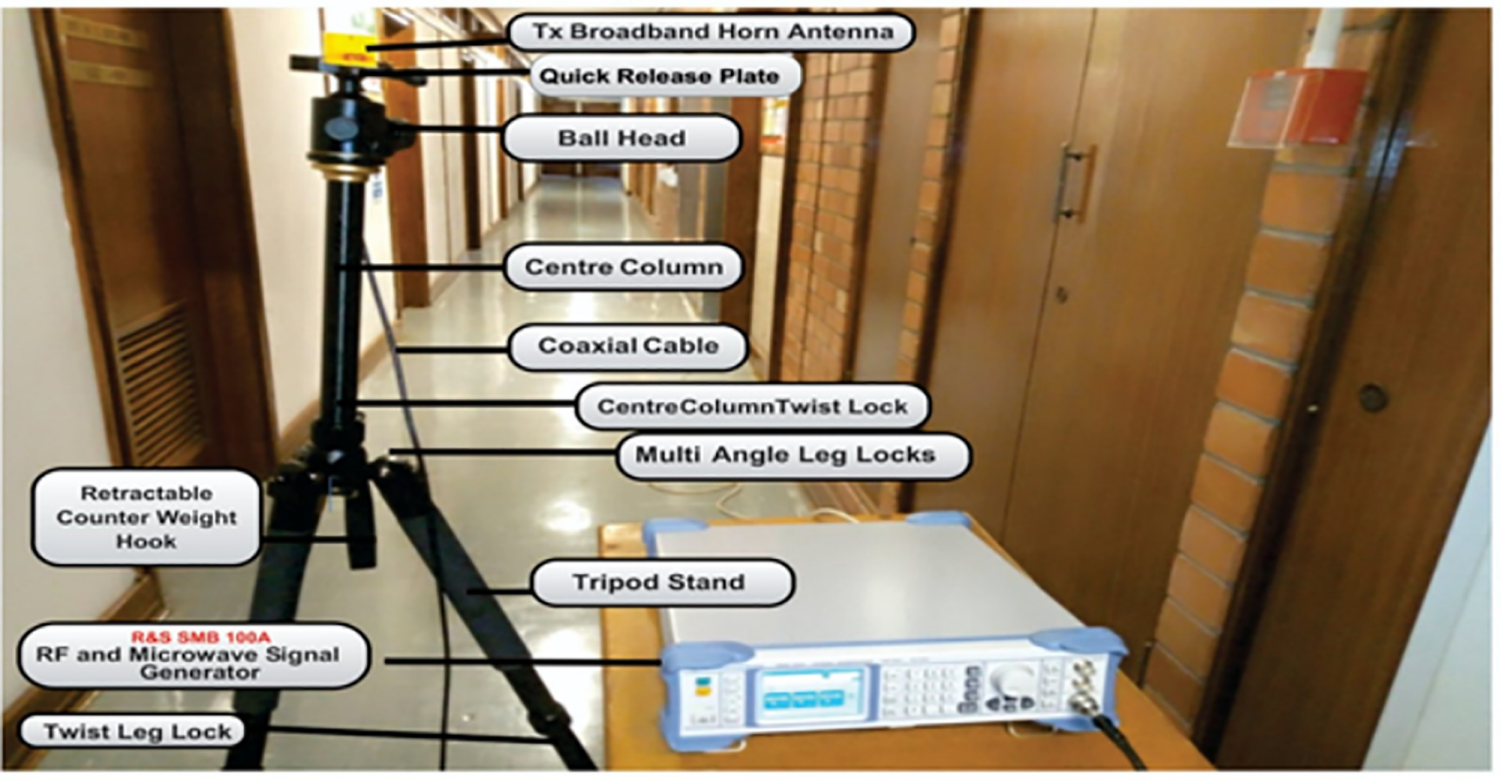
The Tx setup.

**Fig 5:**
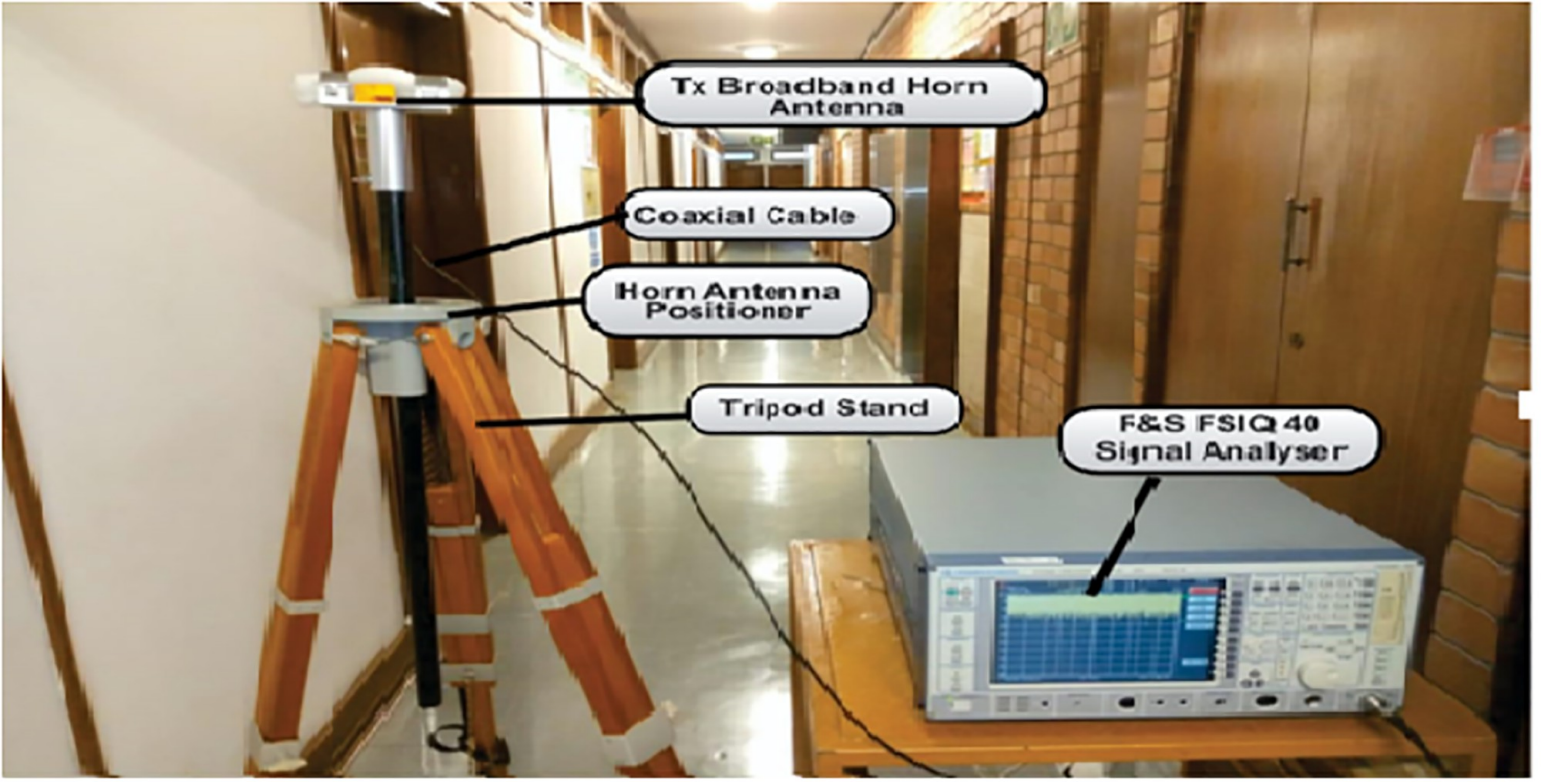
The Rx setup.

**Fig 6:**
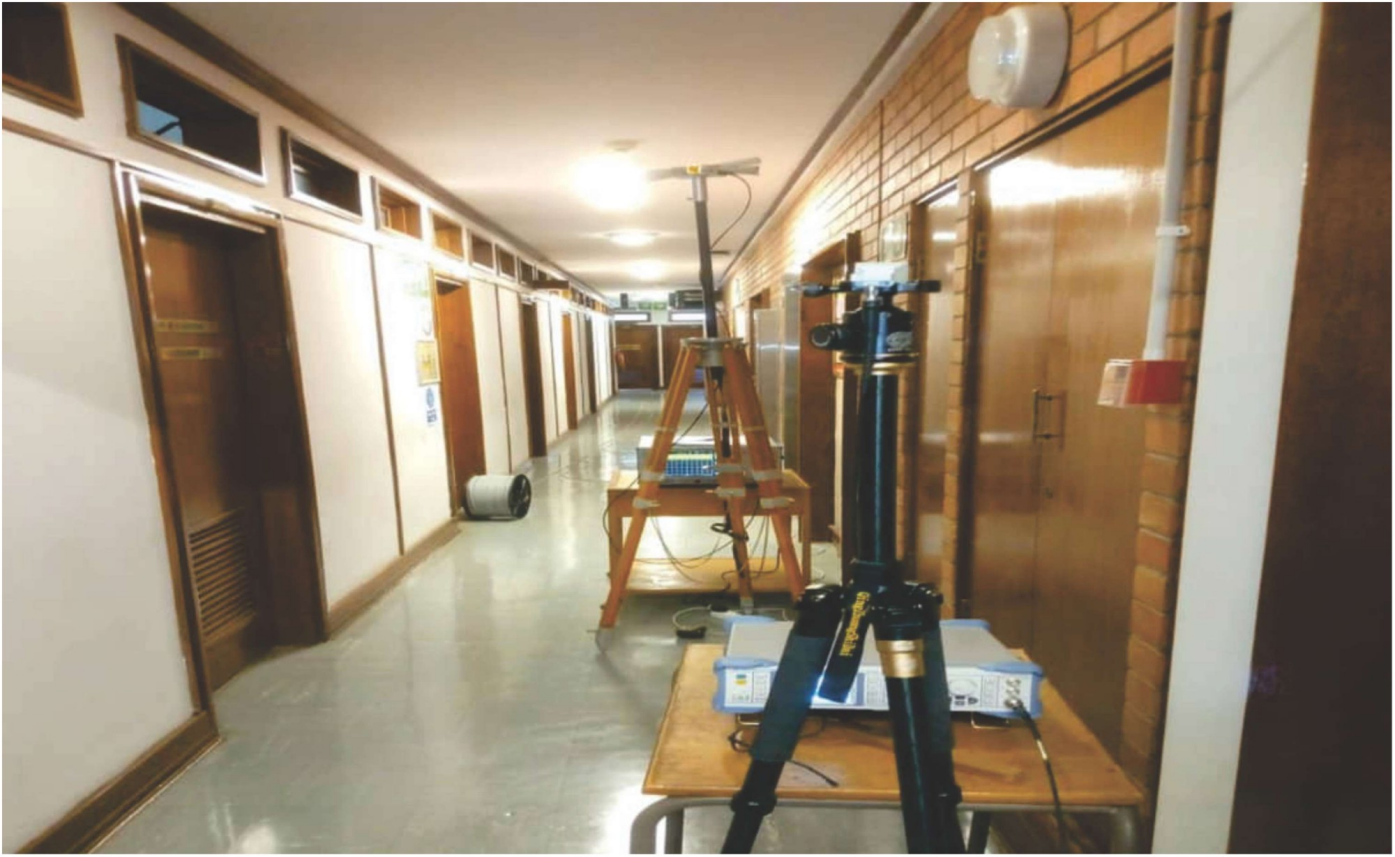
The setup of the Tx and the Rx in the indoor corridor environment.

**Fig 7:**
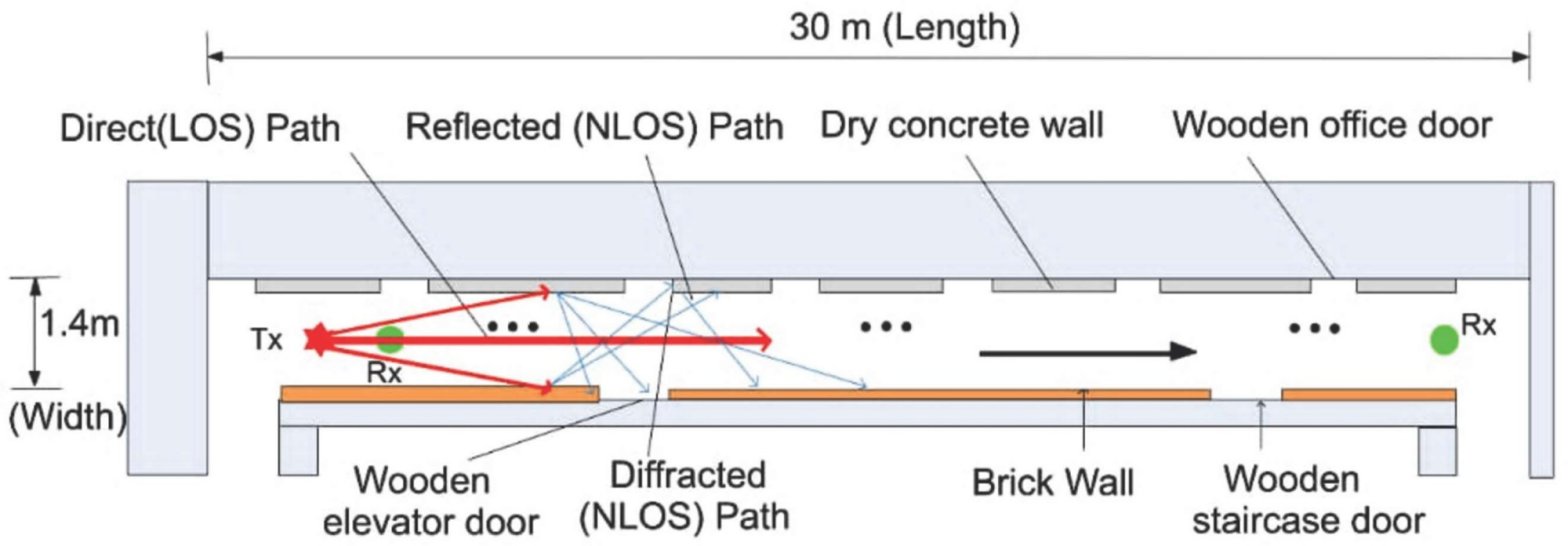
Measurement environment floor plan.

The measurement setup is shown in Fig 2. The Rohde and Schwarz (R&S) 100A SMB radio WS generator was used to provide a continuous wave (CW) signal to the transmitting (Tx) antenna. The receiving system of the set-up has a signal analyzer (R &B FSIQ 40) of a FB range upto 40 GHz. The signal analyzer was connected to a Rx horn antenna (HA) system. The two HAs were used with the FB range of 18 to 40 GHz. Fig 2 provides the detailed explanation for the arrangement. The measurement process involves the LOS as well as the NLOS scenario in V-V and V-H polarizations. The indoor hallway is 30 meters long, 2.63 meters in height and 1.4 meters wide. The Rx HA was shifted away from the Tx by 2 meters at the distance until it reached a distance of 24 meters, while the Tx HA was positioned at one end of the walkway. The LOS situation was assessed with both HA matched on bore sight and no obstructions in their path of the Tx WS. However, in the NLOS there was no alignment between the Tx and Rx HAs. In the meantime, experts in this field recommend a reference distance of 1m between the Tx and the Rx. The number of Tx—Rx separation distances is 13 with a reference distance *d*_*o*_ = 1. The channel sounder’s specifications are listed in Table 1. The detail images for the measurement campaign and the floor plan of the indoor hallway are provided in Figs 3–7. The PL (in dB) is calculated using Eq (1) [32]:

$$P_{L}=P_{t}-P_{r}+G_{r}+G_{t}$$ (1)

where *P*_*t*_, *P*_*r*_, *G*_*t*_, and *G*_*r*_ are the transmitted power, the received power, the gain of Tx HA and the gain of Rx HA, respectively.

**Table 1:** Parameter specifications of the channel sounder.

Parameter	Value/type	Unit
Centre frequency	28, 38	GHz
Transmission bandwidth	100	MHz
Transmission signal	CW	-
Tx and Rx Has	Broadband HA	-
Tx HA power	10	dBm
Tx HA height	1.6	m
Rx HA height	2.3	m
Tx and Rx HA gain at 28 GHz	15	dBi
Tx and Rx HA gain at 38 GHz	17	dBi
Tx and Rx HA polarization	V/ H	-
HA dimension (L x W x H)	71 x 32 x 28.6	mm^3^
HA weight	0.08	Kg

### 3.1. Large scale PL prediction models

Friis’ equation [32, 58, 59] can be used to derive all PL prediction models in general as given below:

$$FSPL\left(f,d\right)\left[dB\right]=10log_{10}\;{\left(\frac{4\pi fd}{c}\right)}^{2}$$ (2)

where *d*, *c* and *f* are the separation distance between the Tx and the Rx, speed of light in the space and the frequency of the WS, respectively. Eq (2) demonstrates that the PL is dependent on the FB and the distance between the Tx and the Rx. The PL formulas are more easily understood on the logarithmic scale. This can be expressed in Eq (3) as follows [32]:

$$FSPL\left(f,d\right)\left[dB\right]=32.4+\;20log_{10}\left(f\right)+\;20log_{10}\;\left(d\right)$$ (3)

The operating frequency in Eq (3) is represented by *f* (in GHz), and f using the expression $10{log}_{10}\;{\left(\frac{4\pi \times 10^{9}}{c}\right)}^{2}$, the value of 32.4 was obtained. The term 20*log*_10_(*f*) is a constant term which is common to the single frequency CI and FI PLMs. Each model has a different representation of this parameter. Let us call this variable as *k*_1_. The PL in free space changes as a square of the Tx-Rx distance and is represented by the expression 20*log*_10_(*d*) which, on a logarithmic scale, is mainly twice the distance. Nevertheless, this number will vary greatly, primarily due to the nature of the medium used to transmit and receive data. It will generally be simpler to designate it as *k*_2_. The PL in terms of *k*_1_ and *k*_2_ is represented by Eq (4) [32].

$$PL\left(d\right)\left[dB\right]=\;k_{1}+\;k_{2}\;\times 10log_{10}\left(d\right)$$ (4)

Where *k*_2_ is a unitless coefficient and *k*_1_ is expressed in decibels. The above equation makes it evident that the WS’s power reduces by $\frac{1}{d^{k_{2}}}$. Therefore, a higher value of *k*_2_ will lead to an increased reliance between the PL and the distance separating the Tx and Rx. According to Eq (1), the SIG strength power at the Rx side is primarily influenced by the *P*_*t*_, *PL*, *G*_*r*_ and *G*_*t*_, as shown in Eq (5) [32]:

$$P_{r}\;\left(d\right)\left[dBm\right]=P_{t}-PL\left(d\right)+G_{r}+G_{t}$$ (5)

### 3.2. CI free space reference distance PL prediction model

The expression of the CI PLM is given in Eq (6) [46]:

$$P_{L}^{CI}\;\left(d\right)\left[dB\right]=FSPL\left(f,d_{o}\right)\left[dB\right]+10.n.\text{log}\left(\frac{d}{d_{o}}\right)+\;X_{\sigma SF}^{CI}$$ (6)

Where $X_{\sigma }^{CI}$, *d*_*o*_, *f* and *n* are the gaussian random variable with zero mean, the reference distance, the frequency of operation and the PLE, respectively. The standard deviation is expressed in decibels (dB). Shadow fading is characterized by widespread changes in PL values caused by obstacles and other random propagation factors [32, 60, 61].

At a reference distance of *d*_*o*_ = 1 *m*, the *FSPL* is expressed in Eq (7) as follows [32]:

$$FSPL\left(f,1m\right)\left[dB\right]=10log_{10}{\left(\frac{4\pi f}{c}\right)}^{2}$$ (7)

Keep in mind that the only significant parameter that needs to be optimized for the CI model is the *PLE* (*n*). This unitless variable indicates how the path loss model is affected by the Tx-Rx gap distance. This model is based on a physical anchor that detects PL close to the transmitting antenna. The transmission frequency found in the FSPL term clearly has a significant impact on the CI model [33, 62]. The CI model’s parameter is enhanced using the mean minimum square error (MMSE) technique. By lowering the SF standard deviation, we can apply this method to minimize the errors while accurately fitting with the measured data.

### 3.3. CI PL prediction model with improvements

The CI model’s equation can be modified by adding an independent component to make the PL prediction more precise and sensitive to the propagation conditions. Two variables in the modified model are dependent on the separation between the Tx and the Rx. The Eq (8) indicates that the PLE concept is presented in the two variables (*n*_1_ and *n*_2_) [32]:

$$P_{L}^{Imp.CI}\;\left(d\right)\left[dB\right]=FSPL\left(f,d_{o}\right)\left[dB\right]+10n_{1}\;log_{10}\left(d\right)+10n_{2}{\left(log_{10}\left(d\right)\right)}^{2}+X_{\sigma SF}^{Imp.CI}\;\text{d}>1\text{m}$$ (8)

Where *n*_1_ and *n*_2_ are the PLE’s first order and second order, respectively. With this CI improvement, it will be easier to suit the actual measured data obtained from the measuring campaigns. The *n*_1_ and *n*_2_ will noticeably fluctuate depending on the environment in which the signal travels. Let’s assume that these parameters have a closed form [32] with *A* = *FSPL* (*f*, *d*_*o*_), *B* = *PL*^*Imp*.*CI*^, *D* = 10*log*_10_ (*d*) and *E* = 10(*log*_10_ (*d*))^2^. Then SF can be written in Eq (9) as:

$$X_{\sigma SF}^{Imp.CI}=B-A-n_{1}D-\;n_{2}E$$ (9)

The experimental data can be used to calculate the SF standard deviation (Imp. CI) as expressed in Eq (10):

$$\sigma^{Imp.CI}=\sqrt{\sum \frac{{\left(X_{\sigma }^{Imp.CI}\right)}^{2}}{N}}$$ (10)

where N denotes the number of Tx-Rx separation distances. We must now distinguish the numerator of Eq (10), with regard to both *n*_1_ and *n*_2_, and set the result equal to zero to obtain the parameters’ ideal values, which will result in the following standard deviation as expressed in Eqs (11) and (12) [32]:

$$\frac{\partial }{\partial n_{1}}\;\left(\sum {\left(B-A-n_{1}D-\;n_{2}E\right)}^{2}\right)=0$$ (11)

$$\frac{\partial }{\partial n_{2}}\left(\sum {\left(B-A-n_{1}D-\;n_{2}E\right)}^{2}\right)=0$$ (12)

The two previous equations have been differentiated and simplified, leaving us with two linear equations that can be written as [32]:

$$\sum D_{n1}^{2}+\sum {\left(DE\right)}_{n2}\;=\sum \left(BD\right)-A\sum D$$ (13)

$$\sum {\left(DE\right)}_{n1}+\sum E_{n2}^{2}\;=\sum \left(BE\right)-A\sum E$$ (14)

The Eqs (13) and (14) can be stated easily in the form of matrix as expressed in Eq (15) as follows [32]:

$$\left[\begin{array}{cc} \sum D^{2} & \sum \left(DE\right)\\ \sum \left(DE\right) & \sum E^{2} \end{array}\right]\left[\begin{array}{c} n_{1}\\ n_{2} \end{array}\right]=\left[\begin{array}{cc} \sum \left(BD\right) & -A\sum D\\ \sum \left(BE\right) & -A\sum E \end{array}\right]$$ (15)

Finally, the earlier matrix can be used to determine the closed-form of *n*_1_ and *n*_2_.

### 3.4. FI path loss prediction model

The two parameters of the FI model are the path loss line slope β and the intercept ∝, as shown in Eq (16) [1, 46]:

$$PL^{FI}\left(d\right)\left[dB\right]=\propto +10\beta log_{10}\left(d\right)+X_{\sigma SF}^{FI}$$ (16)

Where *PL*^*FI*^(*d*) depicts the PL in dB, and $X_{\sigma }^{FI}$ represents the gaussian random variable with a zero mean. The models perform equally well overall in estimating the PL, despite there being a desire for one model over the other depending on the operating FB, environment, and transmitting circumstances of the mobile communication system [10, 32, 62, 63].

### 3.5. FI PL prediction model with improvements

Using the same approach as in the enhanced CI model to add a new variable, the FI model can be improved. This is expressed in Eq (17) [32]:

$$PL^{Imp.FI}\left(d\right)\left[dB\right]=\propto +10\beta_{1}log_{10}\left(d\right)\;+10\beta_{2}log_{10}{\left(d\right)}^{2}+X_{\sigma SF}^{Imp.FI}\;\text{d}>1\text{m}$$ (17)

This model is dependent upon the three parameters i.e. ∝, *β*_1_, and *β*_2_. Using the MMSE process and the identical derivation as for the enhanced CI model, the solution matrix for all these variables is given in Eq (18) [32]:

$$\left[\begin{array}{ccc} N & \sum D & \sum E\\ \sum D & \sum D^{2} & \sum \left(DE\right)\\ \sum E & \sum \left(DE\right) & \sum E^{2} \end{array}\right]\left[\begin{array}{c} \propto \\ \beta_{1}\\ \beta_{2} \end{array}\right]=\left[\begin{array}{c} \sum B\\ \sum \left(BD\right)\\ \sum \left(BE\right) \end{array}\right]$$ (18)

where *B* = *PL*^*Imp*.*CI*^, *D* = 10*log*_10_(*d*) and *E* = 10(*log*_10_(*d*))^2^. Using the prior matrix, the variables’ closed-forms are found. We used actual measurement results of path loss conducted in an enclosed environment to validate the suggested models in Eqs (8) and (17) [32].

## 4. Results and discussion

This section presents the analysis of the study’s findings. The presentation is divided into three segments, each of which illustrates the improved models with the existing ones at 28 GHz as well as 38 GHz. For both V-H and V-V polarizations, the analysis and measurements are given for both LOS and NLOS situations.

### 4.1. Performance analysis of standard CI and improved CI models

**Fig 8:**
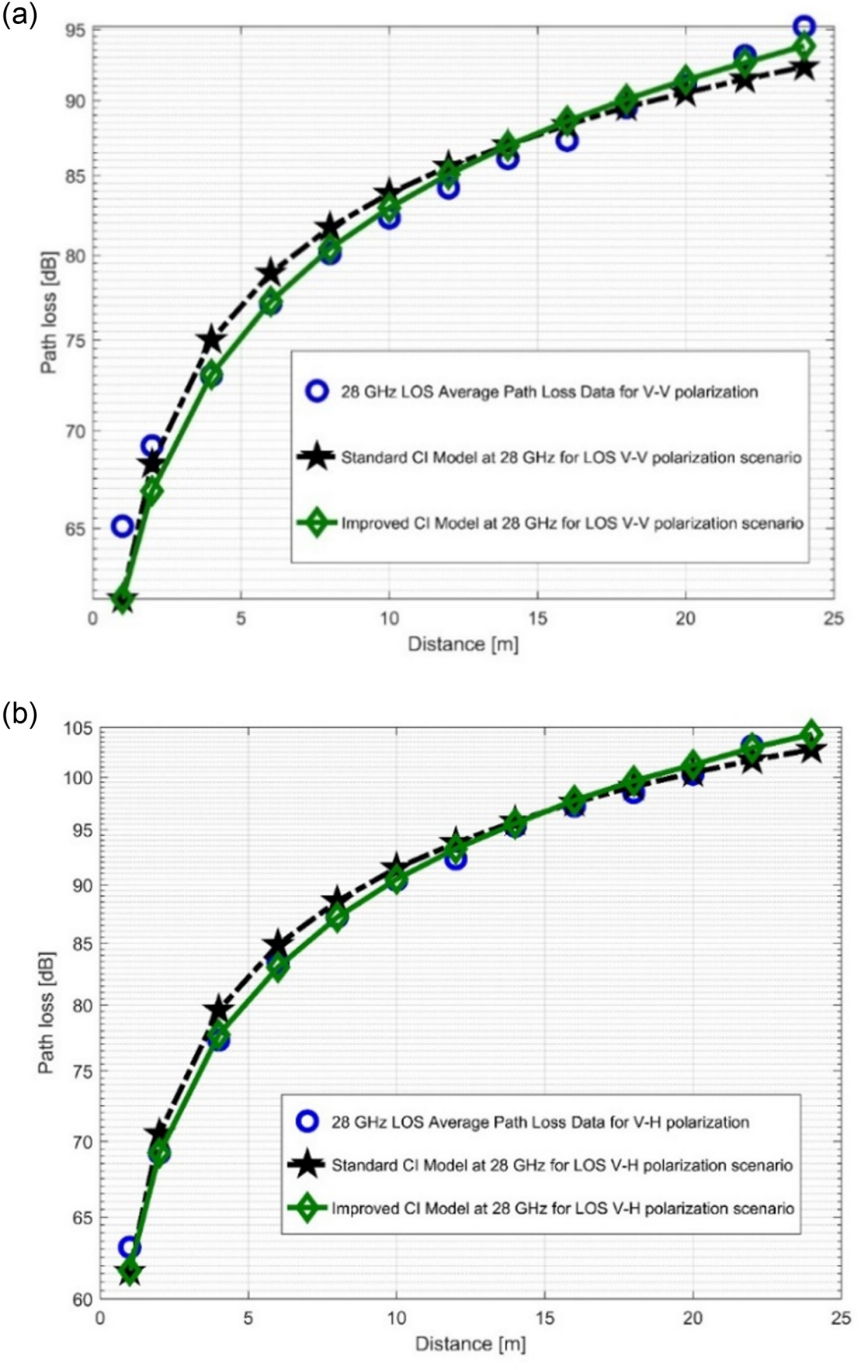
PL versus (vs) distance at 28 GHz for the LOS scenario at (a) V-V polarization, (b) V-H polarization.

**Fig 9:**
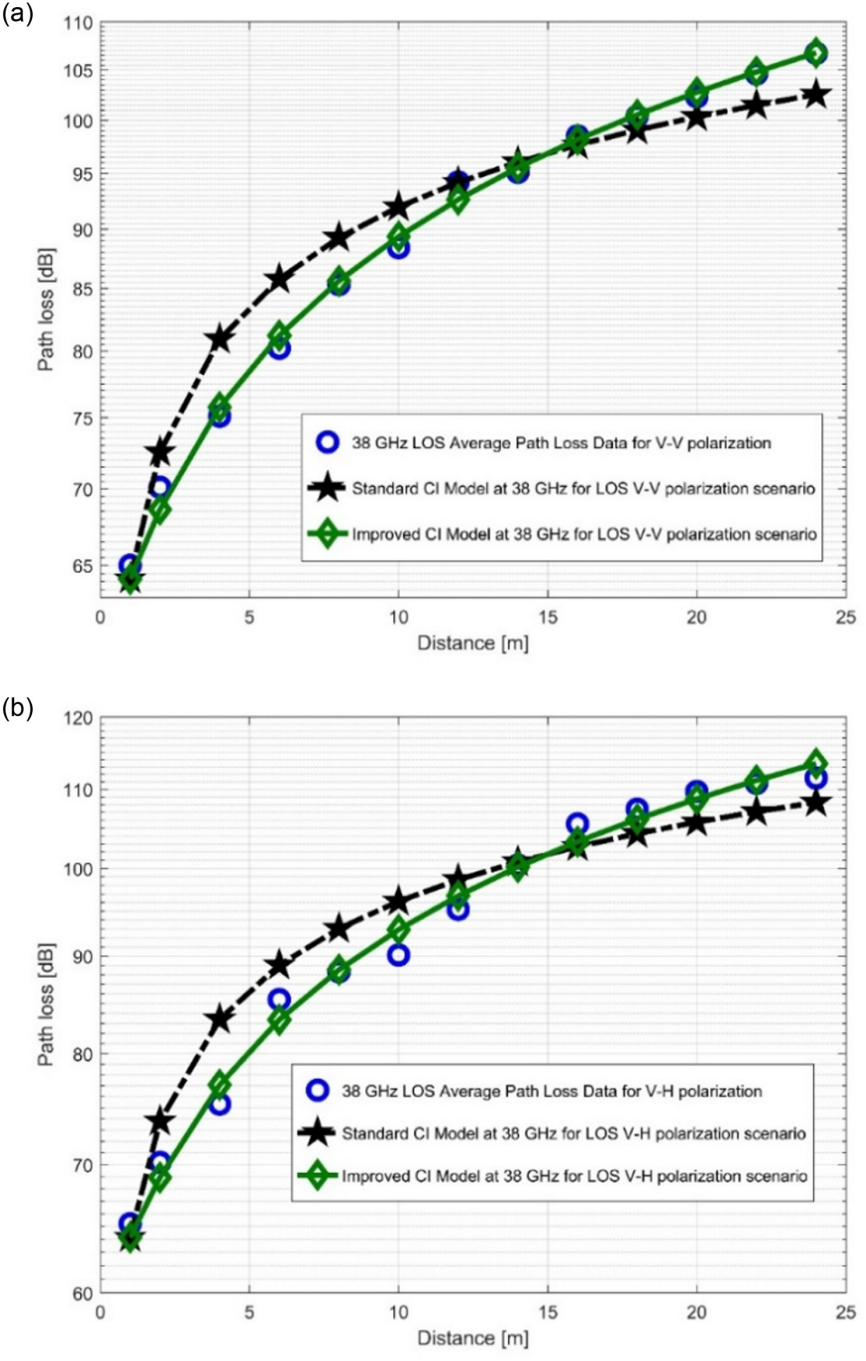
PL vs distance at 38 GHz for the LOS scenario at (a) V-V polarization, (b) V-H polarization.

The path loss analysis for well-known CI model, improved version of the CI model and measurements is plotted at the two FBs in Fig 8a and 8b as well as Fig 9a and 9b. The figures unequivocally demonstrate that the measured PL for the LOS situation is satisfactorily fit by the two models (the improved CI as well as the conventional CI). When antenna polarization is changed, the improved model, which is based on the Table 2 parameters, archives the lower values of the SFSD at both FBs. The reduction of the SFSD for the 28 GHz V-V, 28 GHz V-H, 38 GHz V-V, and 38 GHz V-H FBs are 0.3748 dB, 0.6232 dB, 2.372 dB, and 2.5306 dB, respectively. It should be noted that the standard deviation value decreases as the frequency increases. This was due to an increase in propagation effects and PL. The PLE values increase with the frequency, despite being higher in the V-H polarization. Two more parameters are added to the improved model. These variables are denoted by *n*_1_ and *n*_2_. It is evident that *n*_1_ in both polarizations is greater than *n*_2_ for the 28 GHz FB. The decrease in *n*_2_ was balanced by an increase in *n*_1_ to make the model follow all of the observed PL as well as WS effects. At 38 GHz, there is a different situation where *n*_1_ values are greater than *n*_2_ values. This suggests that the increased PL occurs at higher FBs due to more pronounced propagation effects and WS degeneration. There is a high level of fitting of the improved model to the measured data in Fig 8a and 8b as well as Fig 9a and 9b. This shows that the improved model can predict the PL very well.

**Table 2:** Parameters for the standard CI and improved CI models.

Parameters	V-V polarization (28 GHz)	V-H polarization (28 GHz)	V-V polarization (38 GHz)	V-H polarization (38 GHz)
PLE (*n*)	2.2254	2.9790	2.7801	3.1978
$\sigma_{min}^{CI}$ [dB]	1.7718	1.3425	3.1874	4.1001
$n_{1}^{Imp.\;CI}$	1.6058	2.3345	1.0281	1.0442
$n_{2}^{Imp.\;CI}$	0.5273	0.5485	1.4909	1.8328
$\sigma_{min}^{Imp.\;CI}$ [dB]	1.3970	0.7193	0.8154	1.5695

Both the Tx as well as the Rx HAs been not physically aligned or in line of sight in the NLOS scenario. Only the effects of the corridor environment; diffraction, reflection, and wave guiding, allow WSs to pass from the Tx to the Rx. In contrast to the LOS scenario, PLE values are thus overwhelmingly high. These values are still better compared to the outdoor scenarios with a maximum rate of WS fluctuations. With the CI model, the PLE values are 2.8815 for V-V at 28 GHz band and 3.3303 for V-H at 28 GHz band. Other values of PLE are 2.8207 for V-V at 38 GHz band, 3.4682 for V-V at 38 GHz band and 3.4682 for V-H at 38 GHz band.

The improved version of the CI model responded positively to adjustments in SF standard deviation values in the NLOS scenario. The reductions in the SF standard deviation are 1.6132 dB (at 28 GHz V-V), 2.0917 dB (at 28 GHz V-H), 0.1697 dB (at 38 GHz V-V), and 0.5992 dB (at 38 GHz V-H). With the exception of 28 GHz, which had a higher value for the SFSD for the CI PLM, indicating that 28 GHz is more sensitive to the effects of wireless links than the other FBs under study. Table 3 presents the specific parameters.

**Table 3:** Parameters for the standard CI and improved CI models for the NLOS scenario.

Parameters	V-V polarization (28 GHz)	V-H polarization (28 GHz)	V-V polarization (38 GHz)	V-H polarization (38 GHz)
PLE (*n*) (28 GHz)	2.8815	3.3303	2.8207	3.4682
$\sigma_{min}^{CI}$ [dB]	8.1287	10.4790	1.6822	3.0257
$n_{1}^{Imp.\;CI}$	5.6450	6.9020	3.2393	4.4959
$n_{2}^{Imp.\;CI}$	-2.3517	-3.0396	-0.3562	-0.8746
$\sigma_{min}^{Imp.\;CI}$ [dB]	6.5155	8.3873	1.5125	2.4265

**Fig 10:**
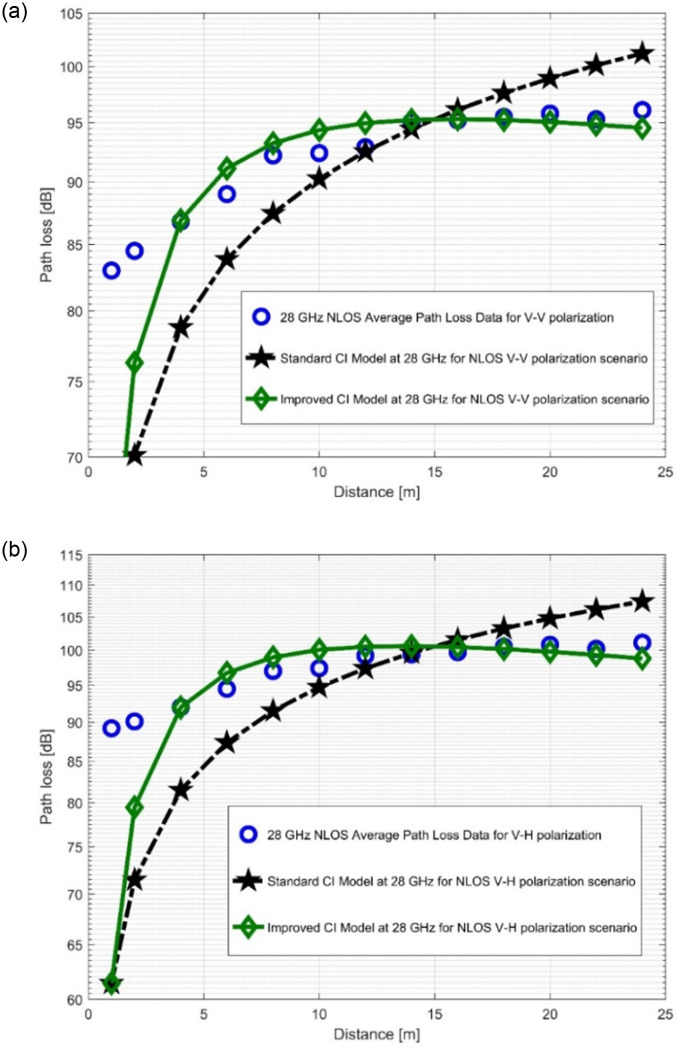
PL vs distance at 28 GHz for the NLOS scenario at (a) V-V polarization, (b) V-H polarization.

**Fig 11:**
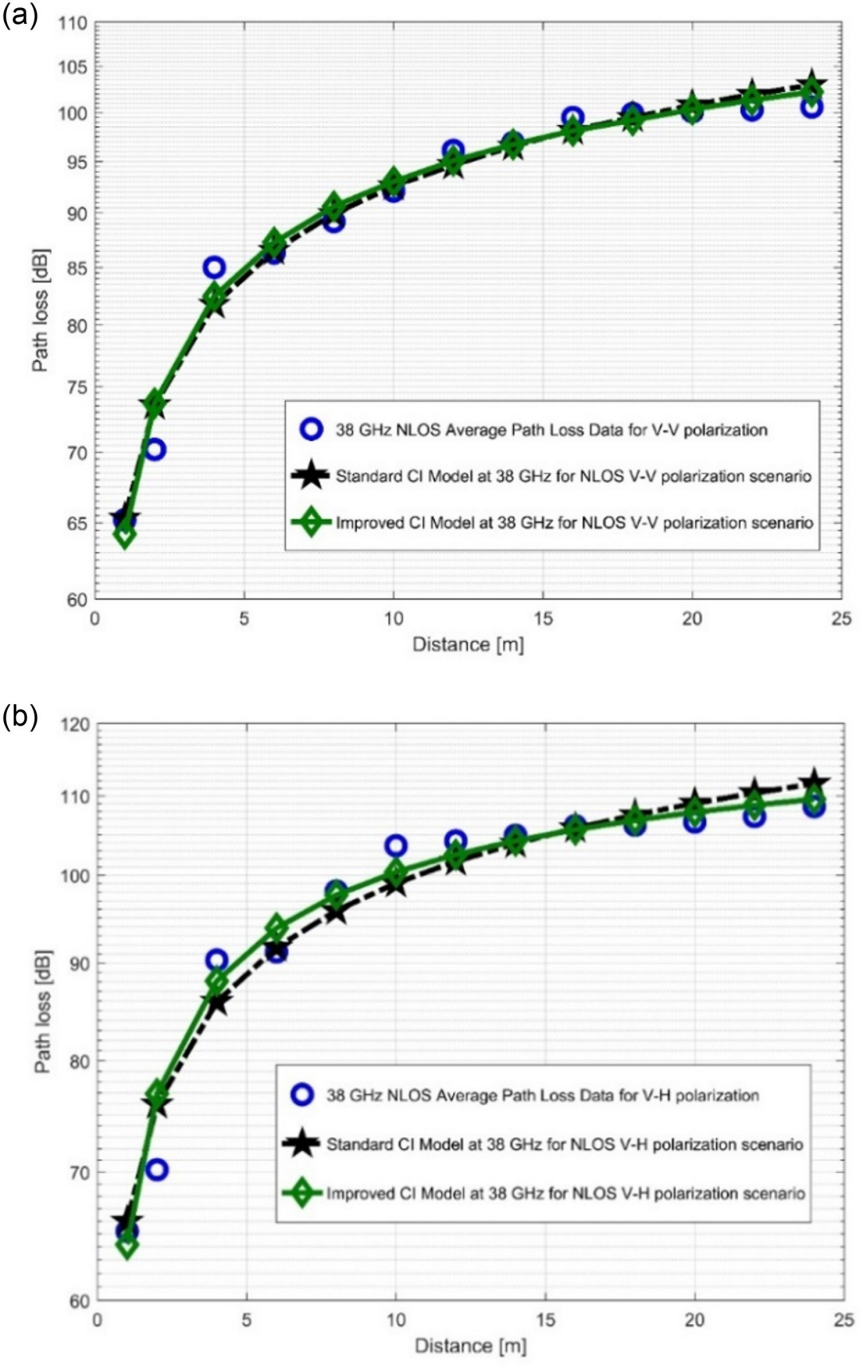
PL vs distance at 38 GHz for the NLOS scenario at (a) V-V polarization, (b) V-H polarization.

Regardless of the polarization, *n*_1_ values are high in both FBs, whereas *n*_2_ values are consistently negative. The model can take into account all potential WS effects in addition to the measured PL because the negative values of *n*_2_ balance out the increase in *n*_1_ values. Fig 10a and 10b, as well as Fig 11a and 11b show how the improved model’s curves differed from those of the current CI model, showing a notable improvement and increased accuracy in PL forecasting. The CI model’s shadow fading standard deviation increases as a result of the NLOS scenario. PL prediction in NLOS situation is therefore less precise than in LOS scenario. The proposed model nevertheless resulted in a SFSD reduction of 0.1697 dB, as shown in Table 3, and offers an appealing decrease in standard deviation. The behavior of the path loss in Fig 10a and 10b as well as in Fig 11a and 11b is understandably not similar with the LOS scenario. The reason was the higher PL experienced in this situation. Nevertheless, the improve model’s performance in the curves (Figs 10a, 10b, 11a and 11b) shows that the improved model is capable of estimating the PL values with respect to the distance accurately.

### 4.2. Performance analysis of standard FI and improved FI models

**Fig 12:**
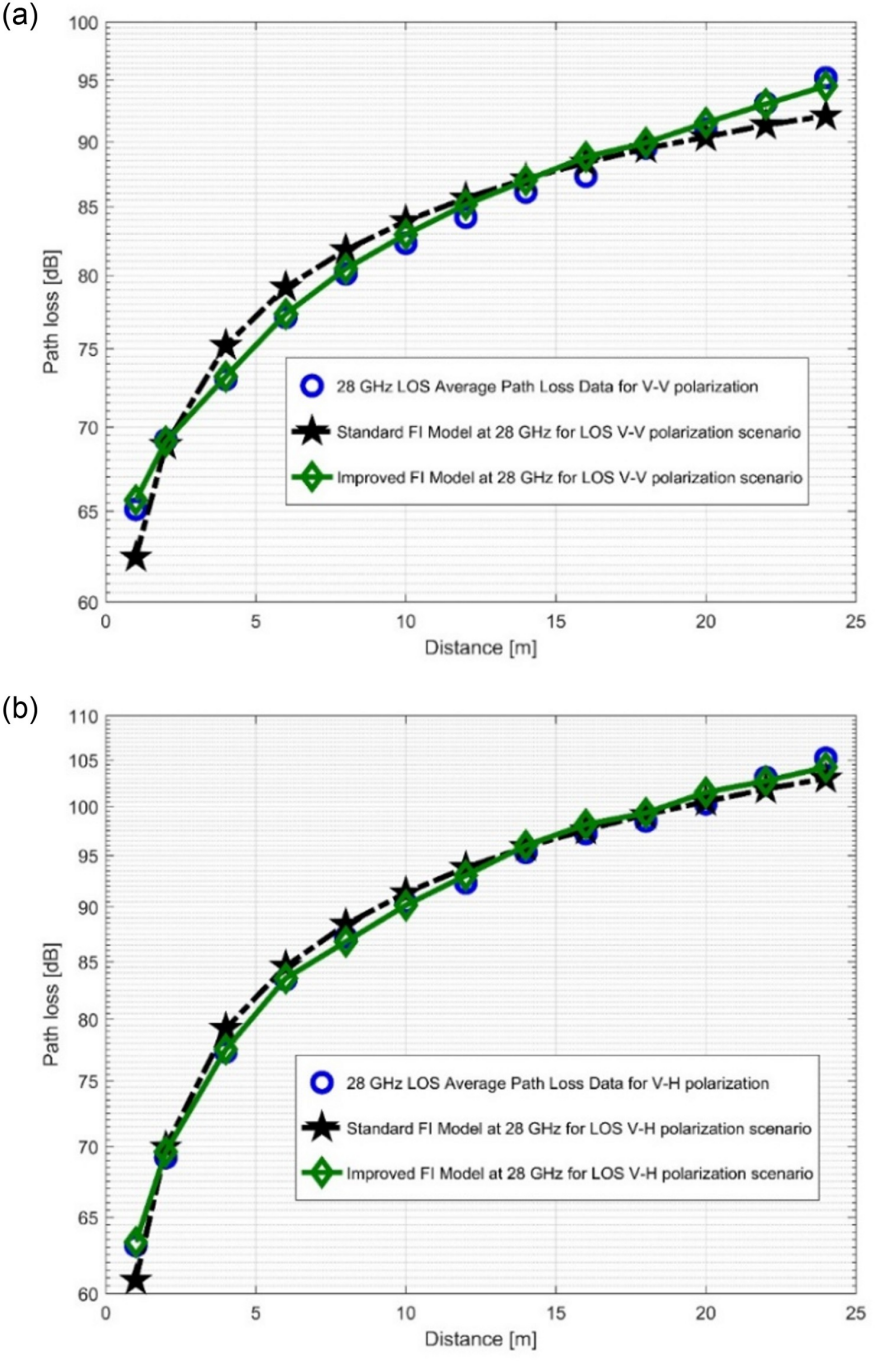
PL vs distance at 28 GHz for the LOS scenario at (a) V-V polarization, (b) V-H polarization.

**Fig 13:**
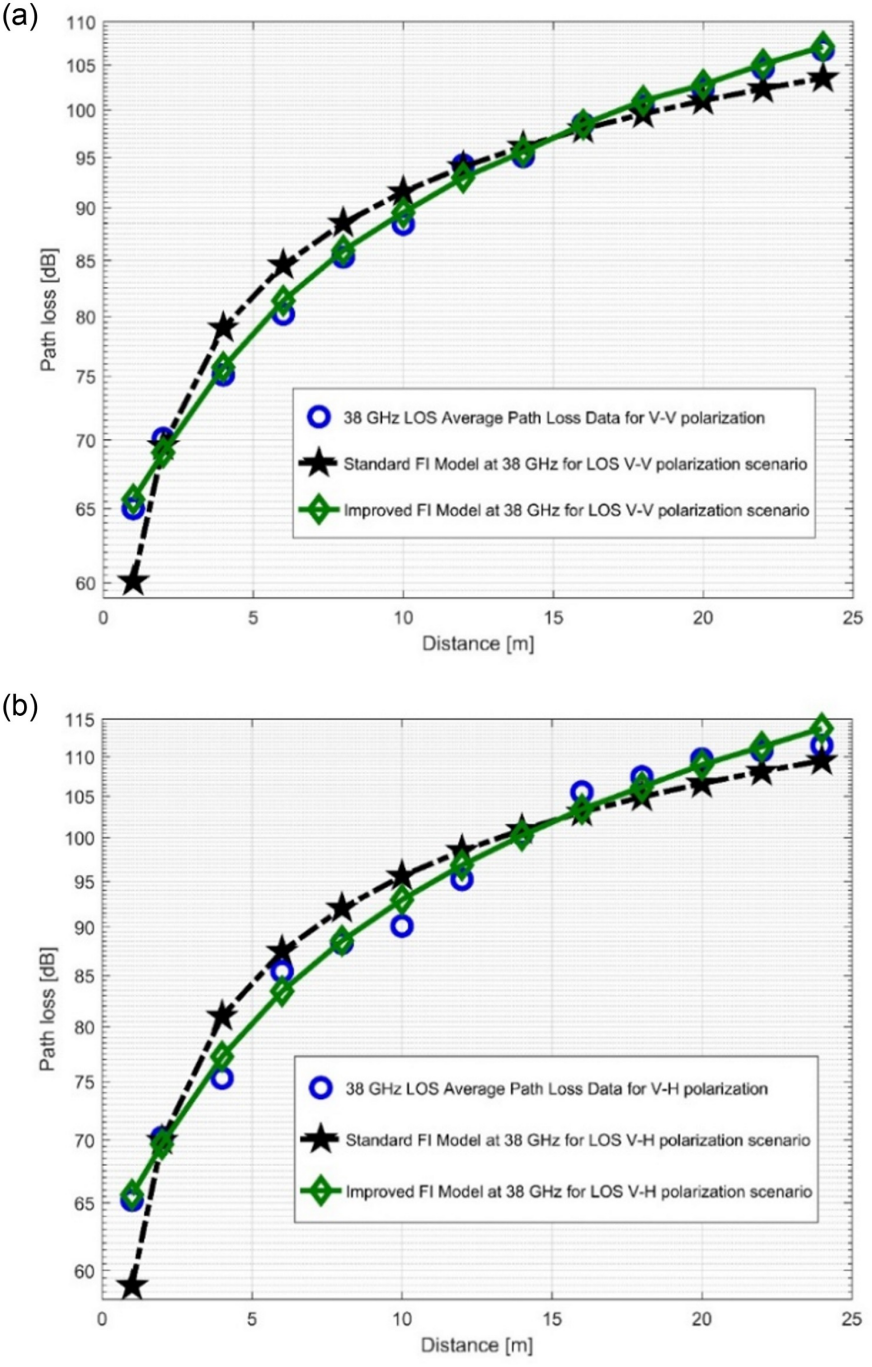
PL vs distance at 38 GHz for the LOS scenario at (a) V-V polarization, (b) V-H polarization.

**Fig 14:**
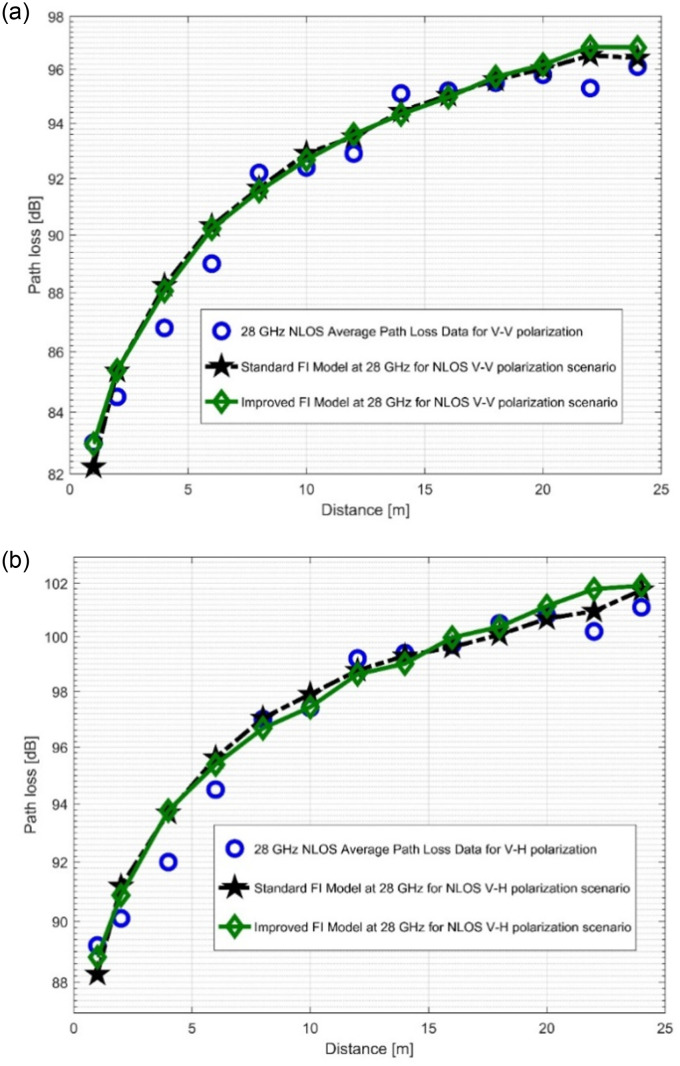
PL vs distance at 28 GHz for the NLOS scenario at (a) V-V polarization, (b) V-H polarization.

**Fig 15:**
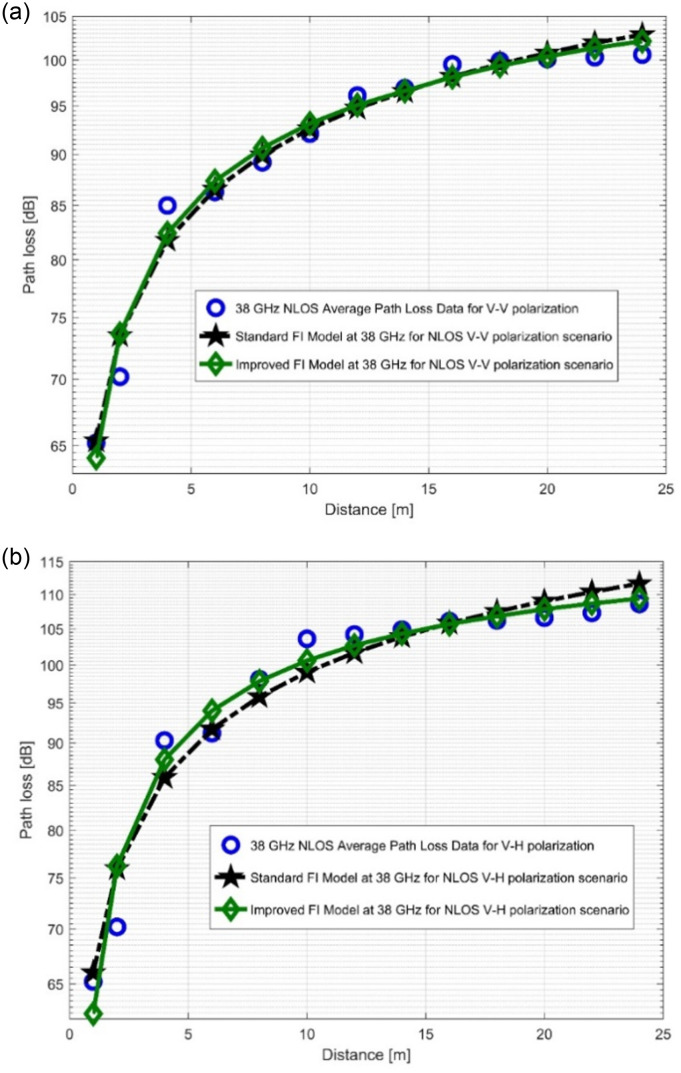
PL vs distance at 38 GHz for the NLOS scenario at (a) V-V polarization, (b) V-H polarization.

The path loss analysis of the well-known FI model, measured values, and improved version of the FI model is presented in Fig 12a and 12b as well as in Fig 13a and 13b. Table 4 displayes the model specifications for the LOS case. The SFSD values in the improved FI PLM appreciably progressed. The improvements of the SFSD are 1.2013 dB (at 28 GHz V-V), 0.7723 dB (at 28 GHz V-H), 2.0379 dB (at 38 GHz V-V), and 2.0307 dB (at 38 GHz V-H). At 38 GHz, the most improvement is seen for V-V polarization. This shows that the improved FI has the highest accuracy for the PL prediction at 38 GHz (V-V). With the improved FI PLM, the floating intercept values have been improved significantly. Fig 14a and 14b, as well as Fig 15a and 15b depict the curves of the recorded FI values and the enhanced FI PLM’s values in the NLOS scenario. In the NLOS situation, the effect of changes in antenna polarizations and FBs remained stable. For the V-H antenna polarization, there is better performance in the shadow fading standard deviation at 38 GHz, with a performance improvement of 0.5735 dB. The slope of the PL curve is divided into *β*_1_ and *β*_2_. The model can keep track of the measured PL and count all potential WS effects due to these two parameters. Therefore, whenever the value of *β*_1_ increases, the value of *β*_2_ always decreases to make up for it. Table 5 displays the specific parameters of the standard FI and improved FI PLMs for the NLOS scenario.

**Table 4:** Parameters for the standard FI and improved FI models for the LOS scenario.

Parameters	V-V polarization (28 GHz)	V-H polarization (28 GHz)	V-V polarization (38 GHz)	V-H polarization (38 GHz)
*α*_*FI*_ [dB]	58.8294	59.9354	60.5444	62.1883
*β*_*FI*_	2.1537	3.0540	3.1461	3.6625
$\sigma_{min}^{FI}$ [dB]	1.7431	1.3008	2.7439	3.5455
*α*_*Imp*. *FI*_ [dB]	65.3730	65.3464	62.9705	65.5702
$\beta_{1}^{Imp.\;FI}$	0.6415	1.9692	0.7265	0.7364
$\beta_{2}^{Imp.\;FI}$	1.0301	0.7390	1.6482	1.9932
$\sigma_{min}^{Imp.\;FI}$ [dB]	0.5418	0.5285	0.7090	1.5148

**Table 5:** Parameters for the standard FI and improved FI models for the NLOS SCE.

Parameter	V-V polarization (28 GHz)	V-H polarization (28 GHz)	V-V polarization (38 GHz)	V-H polarization (38 GHz)
*α*_*FI*_ [dB]	81.8470	87.8146	65.1057	65.8500
*β*_*FI*_	1.0558	0.9722	2.7254	3.3064
$\sigma_{min}^{FI}$ [dB]	0.7872	0.7796	1.6283	2.9396
*α*_*Imp*. *FI*_ [dB]	82.4265	88.5524	63.8704	62.2893
$\beta_{1}^{Imp.\;FI}$	0.7967	0.6423	3.2778	4.8987
$\beta_{2}^{Imp.\;FI}$	0.1765	0.2247	-0.3763	-1.0846
$\sigma_{min}^{Imp.\;FI}$ [dB]	0.7342	0.6908	1.5116	2.3661

### 4.3. MPE and SDE performance of the improved models

The FBs below 6 GHz have up until recently been considered for mobile systems due to their ability to support wide area scope and their simplicity of passage through structures. On the other hand, the cell radius for mmWave bands is much smaller, as the environment within which the WS travels have a major impact on the WS’s ability to spread. Additionally, the mmWave frequency spectrum is greatly impacted by the penetration loss in solid materials. In order to accurately analyze performance and ensure the safe deployment of 5G cellular systems, it is crucial to accurately understand and characterize the behavioral patterns of the mmWave FBs at different interior as well as outdoor locations [1, 10, 26–31]. MPE and SDE are the additional parameters used to assess the model’s efficacy. These two indicators contrast the exact receive data provided during the measurement campaign with the predicted amplitude of the WS provided by the models [64]. According to the following equations, the prediction error (PE) is the difference between the measured and predicted Rx powers which is dependent on the separation of the Tx-Rx. This is expressed in Eq (19) [64].

$$PE\left(d\right)\left[dB\right]=P_{r}^{M}\;\left(d\right)\left[dBm\right]-\;P_{r}^{P}\;\left(d\right)\left[dBm\right]$$ (19)

where ***PE*** (***d***) and $P_{r}^{M}\;\left(d\right)$ represent the prediction error (in dB) and the predicted value of the received power (in dBm), respectively. The expression for $P_{r}^{P}\;\left(d\right)$ is explained further in Eq (20):

$$P_{r}^{P}\;\left(d\right)\left[dBm\right]=P_{t}-P_{L}^{P}\;\left(d\right)\left[dB\right]+G_{r}+G_{t}$$ (20)

Where *P*_*t*_, *G*_*r*_ and *G*_*t*_ are the transmitter power, the gain of the transmitting HA and the gain of the receiving HA, respectively. The models’ PL is predicted by $P_{L}^{P}\;\left(d\right)$. SDE, represented by Eq (21), is a measurement of how far the errors deviate from the MPE value [64]:

$$SDE\;\left[dB\right]=\sqrt{\frac{1}{N}\Sigma_{i=1}^{N}{\left(PE_{i}-MPE\right)}^{2}}$$ (21)

where ***N*** is the number of recorded PL values.

The values of MPE and SDE for 28 and 38 GHz at both antenna polarizations for the LOS situation are given in Table 6. The MPE and SDE values for each parameter in the standard CI, improved CI, standard FI and improved FI models are also compared. Fig 14a and 14b demonstrate that the improved CI performs better at the V-H polarizations at the 28 GHz FB. In a similar vein, the V-H antenna polarization exhibits a good error performance in the improved FI model. The improved CI model for V-V polarization gives 1.9294 dB which is noted for the MPE’s best performance at the 38 GHz FB. The improved FI model provides an increase in the PE value for both polarizations, but not quite to the same extent as of the improved CI model.

**Table 6:** MPE and SDE parameters for the LOS situation.

	MPE [dB]				SDE [dB]			
Polarization	V-V		V-H		V-V		V-H	
Frequency [GHz]	28	38	28	38	28	38	28	38
Standard CI model	1.5211	2.6632	1.1936	3.6772	0.9044	1.8002	0.7028	1.8593
Improved CI model	0.9884	0.7338	0.5687	1.3580	0.8996	0.4217	0.4791	0.7787
Standard FI model	1.5108	2.2633	1.1306	3.0731	0.8566	1.5534	0.6679	1.7711
Improved FI model	0.5037	0.7528	0.5501	1.2607	0.4011	0.3475	0.4097	0.8274

The MPE is a model predictability metric. The level of prediction accuracy is determined by its reduction. In the LOS scenario, the improved CI model predicts the PL with greater precision for both polarizations and FBs. The NLOS scenario reveals a comparable situation, but the PE is significantly higher than in LOS due to the higher PLE values. In the improved models for CI and FI, it is observed that the SDE values for the FBs of 28 GHz and 38 GHz decrease. This is shown in Table 6. However, it is found that as propagation frequency increases, the SDE’s value reduces. For both polarizations, the best improvement of SDE is at 38 GHz. It is worth mentioned that the MPE and SDE values are high in the NLOS scenario. The fact that the Tx as well as Rx HAs been not located in a straight line of sight was the main reason for this. The MPE and SDE values, however, are within a tolerable range at both 28 and 38 GHz. The values of the MPE as well as the SDE for the NLOS situation are listed in Table 7.

**Table 7:** MPE and SDE parameters for the NLOS situation.

	MPE [dB]				SDE [dB]			
Polarization	V-V		V-H		V-V		V-H	
Frequency (GHz)	28	38	28	38	28	38	28	38
Standard CI model	5.6233	1.2904	7.3110	2.5307	5.8432	1.0629	7.4866	1.6403
Improved CI model	3.1018	1.2504	4.0887	1.7985	5.7089	0.8550	7.3067	1.6433
Standard FI model	0.6920	1.2390	0.6325	2.4524	0.5226	1.0647	0.5811	1.6277
Improved FI model	0.7390	1.2163	0.5962	1.8123	0.3806	0.8568	0.4627	1.5247

## 5. Conclusion

In this work, the analysis of the improved versions of the CI and FI PLMs at 28 GHz and 38 GHz has been presented. The MPE and SDE are also used to check for the efficiency of the improved PLMs. To collect the data for the study a measurement campaign was carried out at FBs of 28 and 38GHz. The R&S SMB 100A radio WS generator was used to generate CW signal and that was supplied over a transmission media to the signal analyzer using broadband Tx and Rx HAs. The analysis also focused on the LOS and NLOS scenarios at V-H as well as V-V polarizations. One of the key findings of this work is that the improved PLMs typically perform better in terms of consistency as compared to the current standard models thereby justifying their high accuracy level. The improved CI as well as the FI PLMs showed a significant percentage improvement even at higher FBs and for various antenna polarizations. The MPE and SDE used in percentage error also show how precisely and accurately the improved models predict the PL. After verifying the precision of the improved CI as well as FI models used in this study, the findings also demonstrated that these models’ fundamental use is suitable for LOS and NLOS indoor environments and for various antenna polarizations at millimeter wave frequencies. Also, noteworthy is the fact that the design engineers and researchers will benefit from the values of MPE and SDE in order to make accurate computation of the system design which covers all surroundings as well as connectivity scenarios. The improvement of current PLM parameters should be the main focus of future work, especially for outdoor areas like big shopping malls and cities.
